# Adult Mid Ileo-Ileal Intussusception Secondary to Inflammatory Myofibroblastic Tumor (IMT): A Case Report and Literature Review

**DOI:** 10.7759/cureus.10902

**Published:** 2020-10-11

**Authors:** Muhammad Farhan, Aimen Bibi, Osama Zulfiqar, Muhammad Imran, Zafar Ali

**Affiliations:** 1 General Surgery, Rawalpindi Medical University, Rawalpindi, PAK; 2 General Surgery, City Hospital, Gilgit, PAK; 3 Histopathology, Shifa International Hospital, Islamabad, PAK

**Keywords:** inflammatory myofibroblastic tumor, ileo-ileal intussusception

## Abstract

The intestinal intussusception as a cause of bowel obstruction in adult is a rare finding and is a heralder for an underlying lesion. This is in stark contrast to intestinal intussusception in pediatric population where the etiology is always a primary or benign condition and thus bears different outcomes. We hereby present a case of a young female adult, without the presence of any significant comorbidity. She presented with non-specific symptoms of small bowel intussusception. She was diagnosed as a case of adult intussusception with abdominal ultrasonography. The patient underwent complete resection of the tumor, without any reduction attempts, as the best plausible therapeutic approach. Further, in post-operative evaluation, complete pathological analysis of the resected specimen divulged the presence of inflammatory myofibroblastic tumor (IMT) as the underlying lead point of mid-ileo-ileal intussusception.

## Introduction

Adult intussusception is a rare clinical condition that accounts for only 5% of all cases of intussusception and 1% of the cases of intestinal obstruction [1,2]. The pediatric population always shows primary or benign intussusception, whereas the adult variant always occurs in the background of some organic lesion. The classic triad of symptoms (abdominal pain, vomiting, and bloody stools) as seen in the pediatric population, is found to be scarce in most cases of the adult variant. Nevertheless, adults may present with one or two of the symptoms of the classic triad.

Inflammatory myofibroblastic tumor (IMT) of small gut shows a rare association with intussusception and obstruction. IMT, a soft idiopathic tumor with varied anatomical location, is mostly encountered in the lungs of children and young adults, validating its infrequency in cases of adult intussusception [3]. We hereby elucidate an atypical case of a 23-year-old female patient with an inflammatory myofibroblastic tumor in intussuscepted ileum with special emphasis on diagnosis and therapeutic modalities of adult intussusception.

## Case presentation

**Figure 1:**
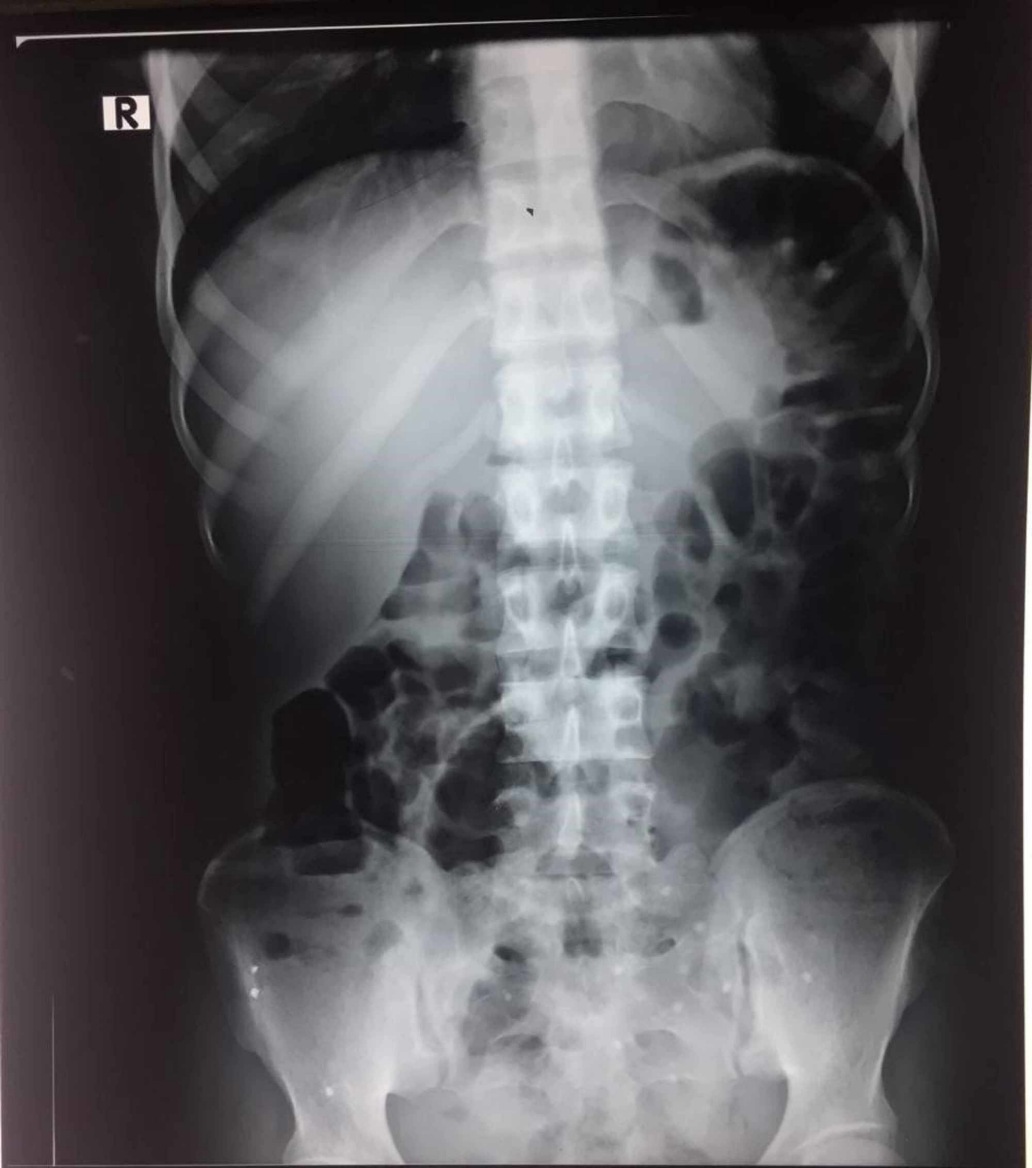
X-ray (abdomen and pelvis) depicting dilated small gut loops.

We present a case of a 23-year-old female with no comorbid conditions. Her prior surgical and medical history was insignificant. She presented to our surgical department with severe abdominal colicky pain, vomiting, and nausea. She also complained of constipation for two days. Her vitals were within normal limits. Physical examination revealed a mildly distended tender abdomen with no enlarged organs or palpable masses. Conservative treatment relieved her abdominal distension and pain. Two days after hospitalization, she again experienced severe abdominal pain with nausea. Her laboratory investigations were: hemoglobin 12.3 g/dl, hematocrit 35.4%, total leukocyte count 17200 μl, serum creatinine 0.3 mg/dl, serum urea 30 mg/dl. Figure 1 shows the plain abdominal radiograph revealing dilated small gut loops in the central abdomen.

**Figure 2:**
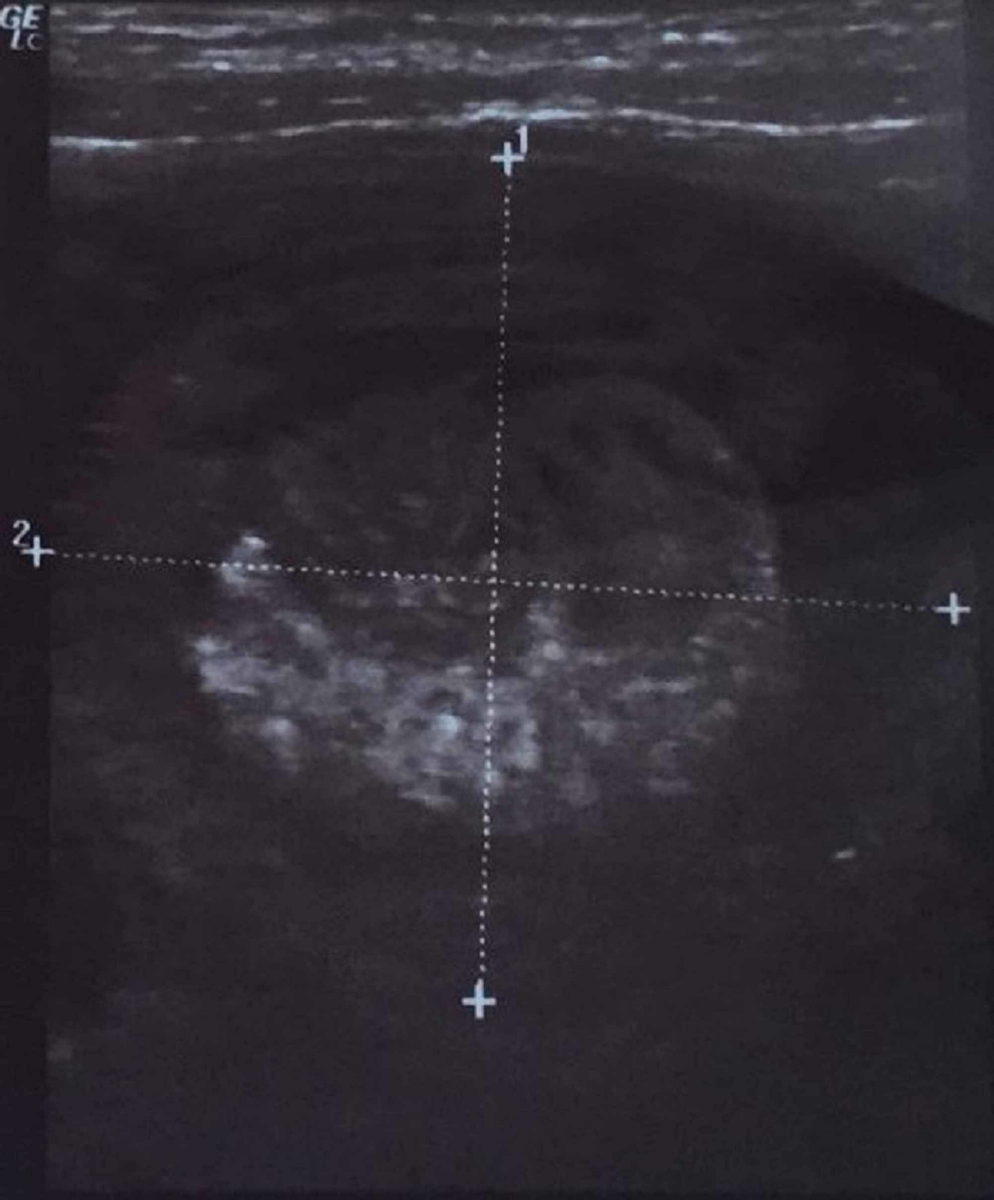
Abdominal transverse ultra-sonographic image showing target type lesion with concentric hypoechoic and hyperechoic layers.

Abdominal ultrasonography revealed a target type lesion with concentric hypoechoic and hyperechoic layers measuring 35 x 38 cm in the lower abdomen with involving guts showing mild color flow and peristalsis, suggestive of intussusception. She was prepared for urgent surgery under diagnosis of intussusception. This is shown in Figure 2.

**Figure 3:**
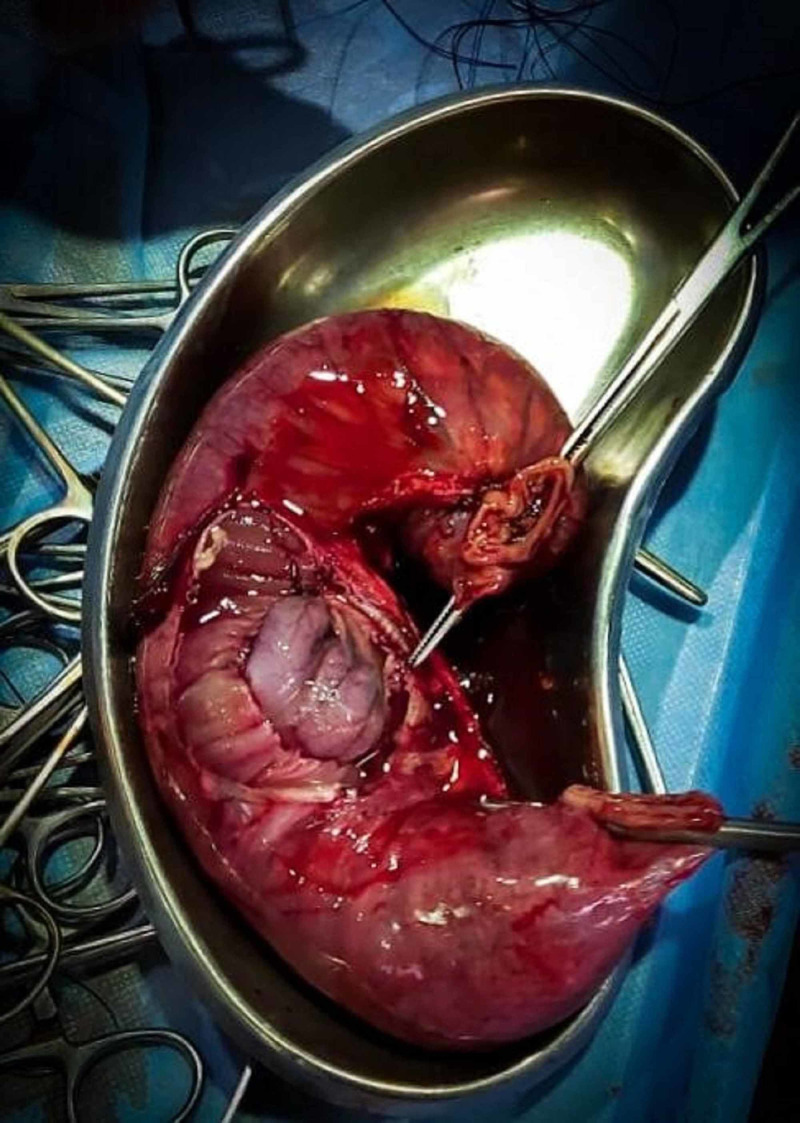
Resected segment of ileum (cut along the greater curvature).

**Figure 4:**
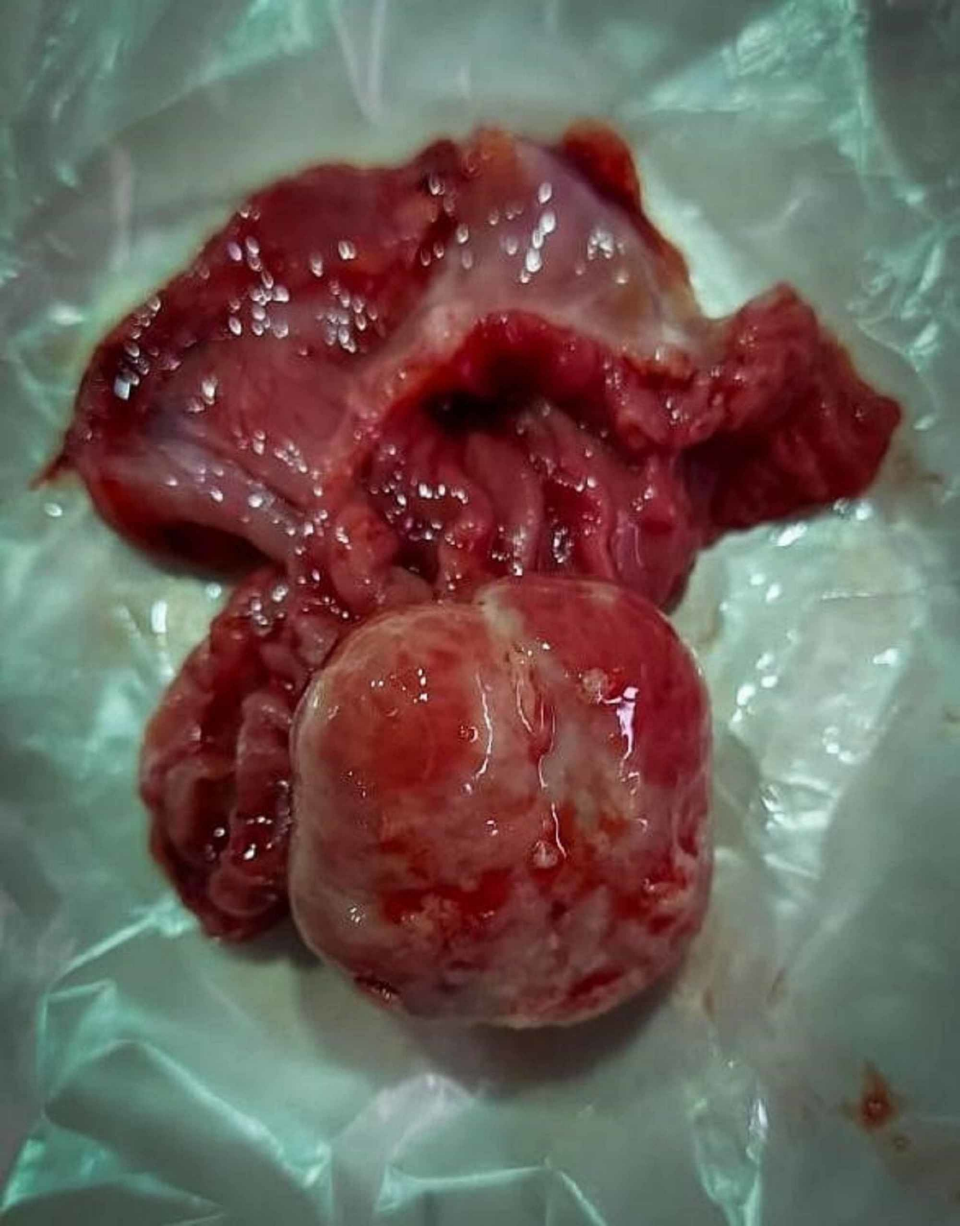
Polypoidal mass taken from the apex of the resected segment of ileum.

During explorative laparotomy, we found mid-ileo-ileal intussusception with proximal gut dilation. Resection of ileal segment and end-to-end anastomosis was performed. No attempt was made for reduction. Grossly, the specimen was 50 cm long with a polypoidal mass of 3.0 x 3.0 cm size at the apex. Figure 3 and Figure 4 show the gross features of the specimen.

**Figure 5:**
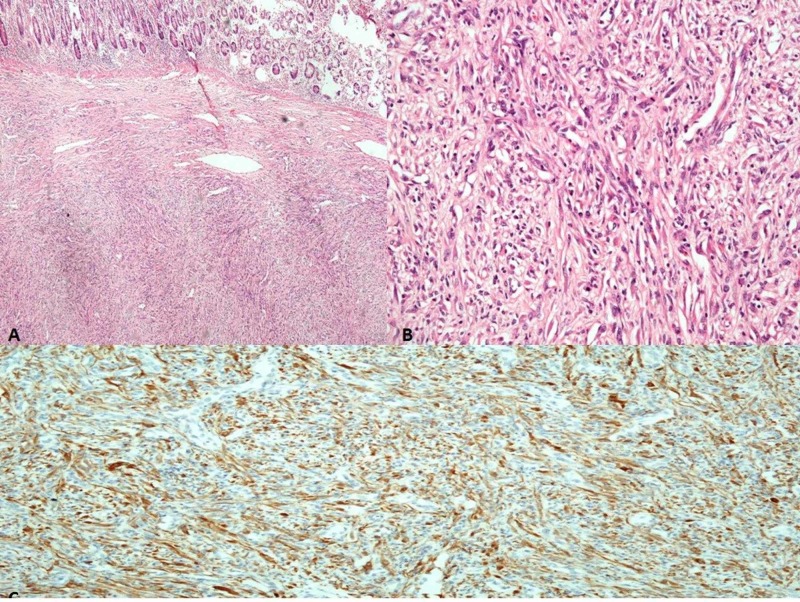
Histopathological evaluation of the specimen. A) Low power view showing spindle cell proliferation (H/E, 200x) B) High power view demonstrating spindled myofibroblast, mature plasma cell, and lymphocytes (H/E, 400x) C) Immunohistochemical staining for anaplastic lymphoma kinase (ALK) in the cytoplasm of tumor cells (H/E, 400x)

Post-operative recovery of the patient was uneventful. Histopathological evaluation of the resected segment divulged a spindle-shaped tumor in the submucosa. These cells were spindle to stellate shaped with infiltration of plasma cells, mast cells, and lymphocytes with immunohistochemistry positive for anaplastic lymphoma kinase (ALK). In the differential diagnosis, inflammatory fibroid polyp was considered; however, the presence of plasma cells and ALK staining is diagnostic of IMT. This is shown in Figure 5.

## Discussion

Intussusception, defined as the telescoping of the proximal segment of the gut within the lumen of the adjacent segment, is a condition found rarely in adults as compared to children with an incidence of two to three cases per population of 1,000,000 per year [4]. It has a significantly varying clinical presentation in adults, unlike the classic triad (abdominal pain, vomiting, and bloody diarrhea) in children. Rather more common symptoms include nausea, vomiting, change in bowel habits, rectal bleeding, and palpable mass but abdominal pain of intermittent and periodic nature and vomiting being the most common of all, creating difficulties in prompt diagnosis [5,6]. As in our case, 23-year-old female patient complained of abdominal pain, vomiting, nausea, and tender abdomen on examination initially.

Pre-operative diagnosis of adult intussusception is difficult because of varying clinical signs and symptoms. Computed tomography (CT) scan is the most sensitive modality for the diagnosis of intussusception with 58-100% accuracy [1]. Abdominal ultrasonography reported to have 35% accuracy, is still considered useful for diagnosis in both resourceful and low resource countries [7]. It is non-invasive with no risk of radiation exposure. Target or Doughnut sign is most commonly demonstrated on a transverse view while on a longitudinal view, we mostly observe hay-fork sign [8,9]. But limitations are there including obesity and bowel gas which may obscure typical findings. So, expertise is required for diagnosis.

While common sites of occurrences are the junctions between freely mobile segments and fixed segments that are retroperitoneal or adhesional [6], our case was found to have mid-ileo-ileal intussusception with proximal gut dilation as indicated by explorative laparotomy. It mostly occurs in small and large gut up to 90% and the remaining 10% occurs in the stomach or surgically created stoma [6]. Adult Intussusception occurs as a cause of an 80-90% underlying lesion with half of them having a malignant etiology in up to 20-60% cases [2,7,9]. Since the incidence of malignancy as the cause of small intestine intussusception, in particular, ranges up to 30%, the case under our study presented IMT as the cause of rare conditioned adult intussusception [10]. The presence of IMT was confirmed by microscopic studies and immunohistochemistry showing positive results for ALK 1 which is found to have overexpression in 8-63% of IMT cases [3]. Furthermore, IMT, being diverse in its histological features with differentiated myofibroblastic spindle cells and accompanied by plasma cells and/or lymphocytes, is found in many organs and soft tissues. IMT shows a wide anatomical distribution, most frequently arising in the abdominal soft tissues, including the mesentery, omentum, retroperitoneum, and pelvis, followed by the lung, mediastinum, head and neck, gastrointestinal tract, and genitourinary tract (including the bladder and uterus). Unusual locations include somatic soft tissues, pancreas, liver, and central nervous system (CNS) [11]. Moreover, extra mesenteric presentation of IMT, especially in ileum causing intussusception is extremely rare [10].

Treatment of the adult intussusception is still under discussion but most surgeons prefer complete resection because of the risk of recurrence and high incidence of malignancies [2, 6]. The gentle reduction is advocated by some authors only in case if any benign etiology is pre-operatively identified [7]. This is in contrast to the therapeutic approach in the pediatric population where reduction is usually done given the absence of any organic etiology. Bearing a small tendency of distant metastasis and local recurrence, resection of the segment believed to be the basic and best therapeutic approach, lies in agreement with our decision of complete resection of the involved segment with end-to-end anastomosis.

## Conclusions

Since the clinical variant of adult intussusception presents with considerable heterogeneity in symptoms, a high level of alertness and appropriate investigations are required for early diagnosis and desired outcomes. Furthermore, IMT should be considered in the differential diagnosis in small intestine tumors. While CT scan appears to be the most sensitive and specific imaging modality, Ultrasonography can also be employed for diagnosis in both emergency and non-emergency situations as observed with the case under our study. Complete resection with end-to-end anastomosis is the most elected approach in adult variants, owing to the presence of an underlying tumor nearly in most cases exempting the need for reduction attempts. Most of these tumors including IMT can only be identified postoperatively by histopathological evaluation.

## References

[1] Gueye ML, Sarr ISS, Gueye MN (2018). Adult ileocecal intussusception induced by adenomatous ileal polyp: case report and literature review. J Surg Case Rep.

[2] Marinis A, Yiallourou A, Samanides L, Dafnios N, Anastasopoulos G, Vassiliou I, Theodosopoulos T (2009). Intussusception of the bowel in adults: a review. World J Gastroenterol.

[3] Waszak M, Sokólska E, Banaszkiewicz Z, Bała A, Korenkiewicz Ł (2015). Inflammatory myofibroblastic tumor within ileal intussusception as the cause of recurrent abdominal pain in a 57-year old patient. Pol Przegl Chir.

[4] Uyulmaz S, Zünd M, Caspar U, Diebold J, Slankamenac K (2018). Ileoileal intussusception in unspecific recurrent abdominal pain in adult: a case report. SAGE Open Med Case Rep.

[5] Honjo H, Mike M, Kusanagi H, Kano N (2015). Adult intussusception: a retrospective review. World J Surg.

[6] Suhaibani YA, Mohamed A, Bhat N, Abukhater M (2009). Adult intussusception, a rare cause of intestinal obstruction, case report and literature review. Internet J Surg.

[7] Gupta V, Doley RP, Subramanya Bharathy KG (2011). Adult intussusception in Northern India. Int J Surg.

[8] Sofia S, Casali A, Bolondi L (2001). Sonographic diagnosis of adult intussusception. Abdom Imaging.

[9] Zubaidi A, Al-Saif F, Silverman R (2006). Adult intussusception: a retrospective review. Dis Colon Rectum.

[10] Ida S, Matsuzaki H, Kawashima S, Watanabe M, Akiyama Y, Baba H (2013). Adult intestinal intussusception caused by an inflammatory myofibroblastic tumor. Case Rep Gastroenterol.

[11] Coffin CM, Watterson J, Priest JR, Dehner LP (1995). Extrapulmonary inflammatory myofibroblastic tumor (inflammatory pseudotumor). A clinicopathologic and immunohistochemical study of 84 cases. Am J Surg Pathol.

